# Managing returns to prison from medium-secure services: qualitative study

**DOI:** 10.1192/bjo.2021.928

**Published:** 2021-06-08

**Authors:** Sarah-Jayne Leonard, Caroline Sanders, Jennifer J. Shaw

**Affiliations:** Offender Health Research Network, Centre for Mental Health and Safety, University of Manchester, UK; NIHR School for Primary Care Research, University of Manchester, UK; Centre for Mental Health and Safety, University of Manchester, UK

**Keywords:** Forensic mental health services, prison mental health, offender pathway, secure services

## Abstract

**Background:**

Little is known about factors that influence discharge decision-making for people admitted to medium-secure services from prison, particularly for those who are returned to prison following treatment.

**Aims:**

To explore the organisational influences on care pathways through medium-secure services for those admitted from prison.

**Method:**

We recruited 24 clinicians via purposive and snowball sampling; 13 shared their experiences via a focus group, and 11 shared their experiences via individual semi-structured interviews. A thematic analysis was conducted, producing three overarching themes: maintenance of throughput and service provision, class of two systems, and desirable and undesirable patients.

**Results:**

Data indicated external factors that direct and, at times, limit clinicians’ pathway decisions, including commissioning criteria and legal status under the Mental Health Act 1983 and within the criminal courts system (i.e. whether on remand or sentenced). These factors also influence how clinicians view the role and function of medium-secure services within the wider forensic mental health system, and therefore the types of patients that are deemed ‘appropriate’ for continued treatment when making discretionary pathway decisions.

**Conclusions:**

There remains a deficit in adequate resources to meet the mental health needs of prisoners who are admitted to medium-secure services. To meet the clinical need of all admissions, criteria for prolonged treatment in medium-secure services needs to be reconsidered, and it is likely that provision for the medium-secure hospital estate will need to increase substantially if effective rehabilitation of those who transfer from prison is to take place.

Secure psychiatric services provide comprehensive, multidisciplinary care and treatment for people with severe mental health problems who require physical and relational security to manage their needs. These services operate at three levels of security: low, medium and high. The focus of this research is on medium-secure services (MSS), designed for those who ‘pose a serious danger to the public’.^1^ These services focus on the assessment and treatment of mental health problems, managing the risk that patients pose to others, and reducing further offending.^2^ Referrals for admission to MSS originate from a number of locations, including general psychiatric hospitals, the secure hospital estate (all secure services), the community and the prison estate.

## Transfer from and return to prison

The prevalence of psychiatric disorders within the UK prison population is high.^3,4^ For those in need of psychiatric in-patient care, transfer to secure mental health services is required to provide treatment and therapeutic intervention.^5–7^ This pathway is for patients deemed to require compulsory treatment, and those for whom appropriate care cannot be given in a prison environment. Transfer to secure services can also be directed by the criminal courts for assessment to inform sentencing decisions (see Supplementary Table 1 available at https://doi.org/10.1192/bjo.2021.928 for part 3 of the Mental Health Act 1983 (MHA)). Factors such as psychiatric diagnoses and patient motivation and engagement are considered when deciding whether an individual is accepted for admission to MSS, resulting in the prioritisation of those with severe mental illness (SMI) over those with a personality disorder.^8^ There are also a range of contextual and relational factors guiding likelihood of admission, including bed availability, quality of relationship with referrers and unit ethos.^9^

Each patient will work through their care pathway, with the aim of discharge to an appropriate destination such as the community, another in-patient service or return to prison. MHA section/legal status will guide discharge options for responsible medical officers (RMOs). For example, criminal courts can sentence a patient who is on remand and direct them to prison, or may impose a hospital treatment order in lieu of a custodial sentence, where eventual discharge will be into the community. Sentenced patients with remaining tariff may be returned to prison by their RMO following treatment, whereas others may receive treatment up until sentence expiration, where their MHA status changes to a notional hospital order (Section 37N MHA) and they remain detained in hospital until they are ready for community discharge.

Return to prison from MSS has increased in frequency over the past two decades.^10,11^ Little is known about factors that may influence care pathway decisions and discharge for those admitted to MSS from prison, but it is evident that those returned to prison are a vulnerable group compared with those discharged into the community.^11,12^ Prison returners display significantly more psychotic symptoms at time of discharge to prison, and have a higher risk of future violence and a lower prevalence of protective factors that mitigate subsequent risks of offending and relapse.^11^ We recently published the first study to establish the circumstances by which MSS patients are returned to prison.^12^ This included court-directed return (17%), sometimes in opposition to clinical opinion, and return directed by the RMO (83%). Many were returned because of treatment completion, to continue their custodial sentence (40%) or await trial (6%), and 9% were returned because the MSS did not detect symptoms that required ongoing hospital detention. However, 28% of patients were returned because they were not engaging with treatment or being too ‘high risk’ to remain detained within the service, 63% of whom had a primary diagnosis of a personality disorder and 30% of whom had a primary diagnosis of a SMI. Return to prison on these grounds was unexpected, given that discharge of non-engaging high-risk patients into the community from MSS would be unacceptable.^12^ It was also observed that 17% of those returned to prison were documented as eligible for parole and/or close to their earliest release date at the time of return. It was unclear why these patients did not remain in MSS until the end of their custodial sentence, to ensure successful transition into the community.

Collectively, these findings indicated that criteria considered for those being returned to prison may be different from criteria for those discharged via a community care pathway. At present, there is a lack of evidence on how and why these decisions are made. To understand discharge decision-making for those admitted from, and returned to prison, the context of constraints in which decision makers operate and the ways in which clinicians perceive these constraints requires exploration. As such, the aim of this study was to gain insight into clinicians’ experiences of receiving, managing and discharging patients who are admitted to MSS from prison.

## Method

### Design

We used a qualitative triangulation methodology involving a focus group and a series of semi-structured individual interviews.^13^ Both methods of data collection were used to gain a comprehensive understanding of managing returns to prison from MSS.

### Ethics

All procedures involving human participants were approved by the North-West England Multi-Site Research Ethics Committee (approval number 09/H1016/126). Verbal consent was also confirmed for the interview/focus group to be audio-recorded at the start of each interview. The authors assert that all procedures contributing to this work comply with the ethical standards of the relevant national and institutional committees on human experimentation and with the Helsinki Declaration of 1975, as revised in 2008.

### Participants, recruitment and setting

Both focus group and individual interview methods were used to explore clinicians’ experiences, and generated data were treated with equal importance. The focus group allowed the team to gain an initial understanding of managing returns to prison from MSS, capturing a range of clinical experiences and interaction on controversial topics. This understanding then guided exploration of individual accounts. The successive individual data enriched the understanding gained from the focus group and added to the rigour of the initial understanding.^13^ The private nature of the individual interviews also provided a more confidential space for discussion of sensitive topics, such as relationships between local prisons/secure units and other healthcare providers. The focus group was conducted with prison and MSS-based psychiatrists, whereas the inclusion of individual interviews allowed us to speak with other professional groups. Data combination allowed for the generation of a coherent and nuanced understanding of clinicians’ experiences of managing and discharging patients who are admitted from prison.

Twenty-four clinicians were interviewed via focus group or individual interview. Characteristics of the participants are presented in Table 1.

**Table 1:** Interviewee and focus group attendees

	Prison-based	MSS-based	Dual-role
Psychiatrists			
Consultant^a^	9	2	6
Junior	1	--	1
Nurses			
Ward-based	–	1	–
Managerial	–	3	–
Social worker	–	–	1

MSS, medium-secure services.

a. One consultant psychiatrist was retired at the time of the interview.

#### Focus group

Focus group attendees were consultant psychiatrists that had experience of working across the remittal care pathway (*n* = 13). Two were MSS-based, seven were prison-based and four were based in both prison and MSS (dual-role). The focus group took place at a 3-day annual meeting of forensic psychiatrists in Glasgow (the Royal College of Psychiatrists’ Forensic Faculty Annual Conference, 2016). Five attendees were contacted in advance of the event, to confirm their attendance. Those contacted directly were individuals identified during our previous study as key clinicians directly involved in remittal to prison within their MSS.^12^ The remaining eight attendees were a convenience sample recruited via advertisements in the conference materials and telephone call during conference preliminaries. None of the attendees worked within the same MSS or prison mental health service (PMHS), which ensured a range of perspectives from different regions. The focus group was conducted over the lunchtime session in one of the breakout seminar rooms.

#### Individual interviews

Individual interviews allowed for the inclusion of participants other than psychiatrists, to gain more insight into the challenges of managing and discharging those admitted from prison. Interviewees included a further six psychiatrists (two were prison-based, one was MSS-based and three were dual-role), four nurses (all MSS-based) and one social worker (dual-role). All participants had experience of working across the remittal care pathway.

Clinicians from all National Health Service MSS and PMHS were eligible to take part. The majority of participants were contacted directly by the research team via email, to invite them to participate (*n* = 7). Those contacted directly were individuals identified during our previous study as key clinicians directly involved in remittal to prison within their MSS.^12^ The remainder were contacted via email by the research team, upon recommendation of participants who had taken part in the interview themselves (*n* = 4) (i.e. snowball sampling). Interviews were conducted in private rooms at the individual's work place. It was anticipated that the nursing perspective would provide more information with regards to the day-to-day management of those admitted from prison, and the social work perspective would produce data with regards to care coordination and aftercare arrangements.

### Materials and procedure

#### Interview guide

An interview guide was developed to facilitative both the focus group and individual interview, based on outstanding areas of interest highlighted in our previous study.^12^ This centred on five main topics: professional background, current role and responsibilities, management and treatment of admissions from prison, returning individuals to prison and positive and negative experiences of the remittal care pathway.

#### Focus group

Participants read the participant information sheet and completed the consent form before the focus group. One author (S.-J.L.) lead the group, with the support of a co-facilitator external to the project, which was audio-recorded with participant consent. Participants introduced themselves and described their role as an ‘icebreaker’ and to foster interaction. Both the co-facilitator and lead researcher had a participatory role within the focus group, to ensure natural discussion.

#### Individual interviews

Travel to the place of work of each interviewee was arranged upon receipt of their consent form, and meetings took place in their office or a clinic room. The study was reintroduced to participants at the start of the interview, to allow an opportunity for outstanding questions. Interviews were audio-recorded, with participants providing written and verbal consent.

### Note-taking

Notes were intermittently made during the focus group and interviews, and reflective notes were made after each meeting. These covered thoughts on key issues highlighted by clinicians. Note sources were collated and saved onto NVivo (version 12 for Windows, QSR International https://www.qsrinternational.com/nvivo-qualitative-data-analysis-software/support-services/nvivo-downloads) as memos, before data transcription. This allowed for a degree of data familiarisation before the formal transcription process.

### Analysis

Each interview and focus group transcription was transcribed verbatim by a member of the research team (S.-J.L.).^14^ Each interview was transcribed before conducting the subsequent interview. All transcripts were checked for accuracy before analysis. Each transcription was uploaded to NVivo (version 12), and saved as an individual source alongside previously uploaded notes. Data were analysed subject to thematic analysis.^15^ This included the following activities: becoming familiar with the data, generating initial codes, searching for themes, reviewing themes, and defining and naming themes. Two members of the research team (S.-J.L. and J.J.S.) initially coded a quarter of all transcripts independently line by line, allowing for inductive themes to emerge from the data, and for cross-checking of coding. The remainder of the transcripts were coded by S.-J.L. All coding and theme development was supported by NVivo (version 12). Verbatim quotes are used to illustrate themes and subthemes, and are presented in Tables 2–4.

**Table 2:** Theme 1: maintenance of throughput and service provision

Theme	Quote
**External priorities and expectations**	
Short turnaround and prison return	‘I think that it's money that is the issues. I think that there's a lot of pressure from commissioners to bring in people from the prison system but we're bed blocked because we have 4 wards in our hospital … I think that in the way that commissioning works, there's 12-week assessment periods and I think that at some point the ICU [intensive care unit] were trying to get people through on 12 weeks’ assessment period from prison. Do the 12 weeks and bounce them straight back. For a while that's kind of how they were getting their money really.’ P1, nurse, MSS-based
	‘The commissioners pressure, a number of times the commissioners have mention that the proportion of discharges back to prison is low and it is an arbitrary statement without any actual regard to the medical condition of the patient.’ FG9, psychiatrist, dual-role
	‘I mean we have received a lot of advice in recent years, and a lot of pressure from commissioners to remit patients to custody, so to treat patients as if they were in the community, so prison is their home, that's where they go back to, so you'd admit, give treatment for the shortest amount of time then discharge and that discharge is back to prisons. I think mental health professionals, me included, have often resisted that. Something doesn't feel quite right about remitting a patient to prison, discharging someone to prison, both ethically, but also if someone is at the end of their sentence, I think we are more successful in terms of reintegrating patients successfully into the community if we hold onto them. But we are being told by commissioners and managers, probably for the last 3 or 4 years, “no, when your episode of care has finished you should remit”. We don't always do that, but that is definitely an economic pressure.’ P9, psychiatrist, dual-role
Bed pressures	‘If he responds very quickly we will probably get him back to prison partly because pressure on beds. So we've got people waiting in prison that we haven't got a bed for, so in some ways while it might be in the patients here best interests to be discharged from here, if they're well and can be managed quite well in prison, you're probably best getting someone who's acutely psychotic in and using the bed that way.’ P1, nurse, MSS-based
	‘I understand why they might want to do that if it's gonna free up a bed for someone who needs an urgent bed but then they go back to prison for a couple of months and just get shown the door and they might not have that support around them in the community. It's worrying. I guess it comes down to pressure on beds and that need to get somebody who is acutely unwell from the prison into a hospital bed.’ P5, nurse, MSS-based
Innovation	‘It works very smoothly and we also set up a special ward in the MSU [medium-secure unit] for prison transfers so that we can, currently the waiting list, we've got 19 people in PRISON waiting for a medium-secure bed – waiting to get transferred out. We have tried to work innovatively, we set up this ward just to manage prisoners and send them back speedily – it varies in the success I suppose.’ FG9, psychiatrist, dual-role
**Prison mental health services provision**	
	Apologies if this sounds controversial, our experience of treatment within the prison system hasn't always been positive. We've gone to see patients and they might have had one dose of their antipsychotic in a week at follow-up, so generally patients stay with us because of that.’ P7, nurse, MSS-based
	‘So, there has always been a personal reluctance probably shared by others, about sending patients to prison but you have to have a cut-off somewhere, and sometimes with patients, one just knows that they will do much better, if they are discharged from hospital where they'll remain within a caring environment. And we organise accommodation and we supervise them in the community, and we organise their aftercare, whereas you are remitting to custody, yes there are in-reach teams, and if it is a local prison we would have links so it's a lot better, but if you are remitting somebody further afield, as used to happen fairly often, you didn't know what services they were going to receive and then it would be more difficult in terms of the offender manager organising the community care, so on that basis I think there was a reluctance to do it. So even though someone was well enough and wouldn't technically require hospital at that point, we would have kept them on. I think we still do to a certain extent, but less so.’ P9, psychiatrist, dual-role

MSS, medium-secure services.

**Table 3:** Theme 2: clash of two systems

Theme	Quote
**Patients on remand and pre-sentenced patients**	
	‘He presented as quite erratic in prison, he was transferred to us for assessment prior to sentencing and as the clinical teams, on all the wards, we felt that he, there was no evidence of any mental illness … it was a unanimous decision the doctors, the social workers, psychiatrists, nurses, you know, really there was nothing that we saw that indicated a paranoid illness and when we went to, we reflected that and he went back to prison but when he was sentenced, the other expert witness, they felt that he was, that he was unwell and had paranoid schizophrenia and he ended up getting a 37/41 [Section of Mental Health Act]. So he came back to us and that's been very difficult because it's been a decision that isn't entirely comfortable with the clinical team.’ P1, nurse, MSS-based
	‘I do know cases where judges have not been comfortable giving a restrictive order because they don't think that it is restrictive enough. And they've actually said, “well I think this patient should have a life sentence instead” and that's what they've done, so I'm aware of that happening and I know of cases here where that has happened. And then there's been a lot of argy bargy about what should happen next, and often the patient have had to go to prison, at least for a while and then been brought back again.’ P3, psychiatrist, MSS-based
**Patients with a custodial sentence**	
Lengthy sentence	‘One of them's got quite a long sentence so the chances are he will go back to prison just because if he makes good progress we couldn't justify keeping him here for another few years.’ P1, nurse, MSS-based
	‘And I think sometimes if they've got a long tariff and the task from the very beginning is – they'll come to hospital, get treated, and we'll send them back. That decision is usually made early on with a lot of people because it's just about getting them back to prison, getting them treated and getting them on a regime.’ P6, social worker, dual-role
	‘I think for his sake, you know, it's a better experience I supposed sending back to jail you know, but unfortunately he just reaches transfer back – one of the guys who I'm thinking of even though he was settled, there were no problems, he just became unwell again so we had to transfer him back to a hospital, hopefully he'll settle down and we'll send him back out again.’ FG9, psychiatrist, dual-role
	‘So in all likelihood they'll be referred back again in a worse state than they left you in due course, so no one has benefitted from that. And I think this is one of these cases that I've had that have been repeatedly transferred to and fro but that's been rather different, but this has been a case of someone who has got better in hospital, has been returned back to prison and has stopped treatment and the fairly typical revolving door pattern. And I think sometimes you have to take the view of saying, although in theory they should send them back, we know it's going to fail so there's no point in doing it.’ P3, psychiatrist, MSS-based
Release date approaching	‘Actually they will be back out into the community quicker than what they would be if they stayed with us. We have had quite a few patients, probably over, I've managed [redacted] ward for maybe about 3 and a half years now, where they went back to prison and are back out into the community where if they'd stayed with us and went to our rehab ward they would have been in the system longer.’ P7, nurse, MSS-based
	‘Sometimes that's actually what they want to do. You know if they've got a tariff dates or a date of release sometimes it's beneficial for them that they want to go back… We can have individuals who are adamant that they just wanna go back to prison to the point that their behaviour will deteriorate if they think it's gonna get them back quicker. So obviously we will say “right fine” and we will try and get them back as quickly as we can – that might be the next day if the behaviour is deteriorated that badly.’ P5, nurse, MSS-based
	‘And if they were towards the end of their sentence we would almost certainly keep them, because they are only under the Mental Health Act in hospital and you can assure people comply with medication, and if we know that that is crucial to maintaining health then we would keep that person longer.’ P9, psychiatrist, dual-role
	‘I wouldn't say that is a practice that we do because that would – not setting people up to fail, but is that really giving them the support for when they get into the community? I understand why they might want to do that if it's gonna free up a bed for someone who needs an urgent bed but then they go back to prison for a couple of months and just get shown the door and they might not have that support around them in the community – and they're more likely to get that support when they're coming through the healthcare system… I would certainly think that there would be more reoffending or more distress for that individual if they just went back to prison and went straight out and didn't have the support – if we discharge through here into the appropriate housing and accommodation… whereas if that person had just gone back to prison they would be out of that loop.’ P5, nurse, MSS-based
**Patients with an indeterminate sentence for public protection**	
	‘And we have had patients in the unit, who have been ready and we would have judged them to be ready to go to the community then the parole board has come along and said no. And under those circumstances they have had to be remitted to custody even though we would have kept them because only in a custodial setting would everyone, the offender manager, offender supervisor and parole board be satisfied that they have completed the prescribed offending behaviour programmes, so that has happened on occasions.’ P9, psychiatrist, dual-role
	It's terrible, I mean hopelessness is the right word, in the case I'm thinking of, it feels like that and really, if it were up to me I'd discharge him from hospital tomorrow because, you know, there are risks, but there are and I suppose you get into all this trouble of this whole moral thing of what's right and wrong and whether these sentences are morally right and you know that a lot of people have got off on appeals. But also I've discovered that legal aided barristers aren't interested in taking the appeal cases for IPPs [imprisonments for public protection] on because they don't think it's worth their while. But privately, if you can fund your own case, you've got a pretty good chance of getting them off at appeal. If you can't fund your own case you haven't because the barristers just won't take them on. And that feels a bit unjust as well.’ P3, psychiatrist, MSS-based

MSS, medium-secure services.

**Table 4:** Theme 3: desirable and undesirable patients

Theme	Quote
**Forensic patients versus acutely unwell prisoners**	
Culpability and deservedness	‘I suppose if their psychotic state is linked to their offending then I think the best route for them would be out through the secure unit so then they could have all of the appropriate follow-up and the stuff from that… if someone is clearly shown to offend because of their psychotic state and then you've just released them back to prison then I would say that's potentially dangerous and not good care.’ P8, psychiatrist, dual-role
	‘I think it's “well actually they're bad so they should be in prison, but if somebody's mad then they should be in hospital”. And I think there's still that there. I don't think it's really obvert and I don't think it's a gross problem but I think it is there for some people, which I think can determine the way that someone is managed… I think particularly if we have had patients who have come to us where it provokes a certain feeling for people that it has caused difficulties within the team dynamic because somebody might have offended away from mental illness but then they go to prison and then become unwell in prison, and that provokes a different set of challenges for staff… and it's really, because then we have other people who might have come to us and, you know might have stabbed somebody or whatever and it doesn't provoke the same response… And then people, staff can find that easier to manage and to deal with rather than patient who just commits lots and lots of crimes of varying degrees, goes to prison and then becomes unwell in prison.’ P7, nurse, MSS-based
Acutely unwell prisoners and patient view	‘Forensic populations aren't new populations, they don't come out of nowhere, they are often general adult patients who have gone on to offend… There is an argument that forensic services shouldn't take over patients who would ordinarily come under the general psychiatry, so if a person had become unwell at a later, or another point in their pathway, they would be a general psychiatry patient. And there would be no question of them coming to forensic services, just because they happen to become unwell in prison, so if there aren't forensic issues then, they'd be looked after by our general adult psychiatry colleagues.’ P9, psychiatrist, dual-role
	‘First of all he really didn't want to stay in hospital, so that was one big thing. He didn't want to be labelled as a nutter. So he didn't mind being a bit “off his face” as he put it and going back to prison. Being discharged from prison is very different from being discharged from mental institute as they see it, that's just how they see it. It's still quite cool to be released from prison …I suppose the pathway options are built around response, because a lot of them do see themselves as prisoners and want to go back to complete their sentence and move on’ P1, nurse, MSS-based
Risk responsibility	‘Of course, with a hospital order we've got both bits of it then because we're saying we think their risk and their mental illness are closely related. Whereas, with transferred prisoners that's completely different.’ P3, psychiatrist, MSS-based
	‘6 months is probably enough time to get medication and all that under control and then it's about what further input you can provide. Obviously if you're looking to release someone from or discharge someone from this unit not only do they have to be on medication but they have to have all of the other appropriate support and index offence work and that kind of stuff as well so inevitably that would take longer whereas if it's someone who has, whose offences aren't necessarily related to their mental state and it's just a question of, you know, they're a prisoner they've developed a mental illness under a mental health act, once that's treated they can go back again.’ P8, psychiatrist, dual-role
Risk containment and consequences	‘I'd be more comfortable sending someone to prison than a community placement, one because you could argue that the same risk may be there, but it's contained in prison, and you're not, I suppose it's easier because they go, and if they get a service or not it's not really your fault if they do or they don't; you make your recommendations that they do get something.’ P10, nurse, MSS-based
	‘So we get people and we send people back who are still at extremely high risk of violence to others that they wouldn't be discharged on those grounds alone. So you'd be so frightened they'd do something horrible so you'd never send them back to the community, because you couldn't, but because they're sending them back to prison they're not worried about risk. So that risk doesn't seem to come into it, so we get people coming in who are at really high risk of violence to others which is okay, I suppose in terms of that you can manage that in prison but I'm also thinking about release and I'm thinking, what are we gonna do they're going to be released in 6 months’ time, and they're still high risk and it does relate to their mental illness. So what would you like me to do, so what should we do about that? … we just see people coming back all the time, into prison. You know, they're released and then they're back in a fortnight, so you know that they've done something.’ P4, psychiatrist, prison-based
Criticism of current practice	‘I'm going to have a view about that which is, medium-secure units were built for a number of reasons, one of which was to help prisons. Core functions of medium-secure units is to be there for prisoners and to help acutely mentally disordered prisoners in time of need… so some medium-secure units in a few areas have no prisons anymore in their patch or that they serve and I think that over time that leads to a sort of misplaced view of what they're there to do, so they become self-serving rather than serving the prisons that they're meant to serve actually. So they start to see their own functions as the most important one rather than serving the needs of the populations.’ P2, psychiatrist, prison-based
**Nature of patient presentation**	
Non-adherence and disengagement	‘For someone to successfully stay in medium security as a prisoner-patient they will be – they won't be a management problem. So they will affectively go along with the rules and take their tablets and do all those things, go to the groups, participate in treatment. It's the ones that don't do that who go back.’ P1, nurse, MSS-based
	‘And the other thing is, I've had people that essentially you're only bringing them in for medication as you can't medicate them in prison, they don't engage in anything here, they don't do the psychology, they're not getting any benefit apart from -you can medicate them so if you get to a point where they're taking their medication, their symptoms have gone but actually they're just doing nothing apart from staying in their bedroom all day – they can do that in prison, you don't feel that they're actually benefitting from the regime or what's on offer here.’ P11, psychiatrist, dual-role
	‘So they just weren't engaging with the rehab – off you go. We couldn't do anything with them; they were taking up important, valuable beds so they went back to prison… we're not gonna have somebody here who's not gonna take an opportunity, grab that chance while they can – no, we'll get you out and we'll get somebody in who wants that chance. And if they don't want that chance as well, then we'll send them back. There's a waiting list a mile long to come here so go back.’ P6, social worker, dual-role
Ward-based risk	‘Again he was someone who wasn't, I think there certainly was the will from our team to try and engage him in treatment but he just wasn't in a place to be able to do so unfortunately so again there wasn't too much more we could offer him at the stage and he wasn't really engaging in any therapy and was subverting security and making the area unsafe and we thought “if he's not able to engage in such therapies, he's someone who could potentially get stuck in the system”, so he was again transferred back to prison.’ P8, psychiatrist, dual-role
	‘I think that what tends to happen is if they display difficult behaviours, if they push the system if they are violent if they become assaultive – any of those things, then the first thing that will be said is that they should go back to prison. So that's, I think people are just very quick to wash their hands of them, it's “this isn't our problem, we're not paid enough for this, we don't know how to work with people like this”, it's that kind of this really… I think that when you're full time and you're in the ward and you're getting battered every day and you think that someone is in control of their behaviours and they're already on a prison sentence, I think the temptation can be a lot more that they, you know, they're already being dealt with, they're a write off just bounce them back to prison.’ P1, nurse, MSS-based
Criticism of current practice	‘The trouble is, we have had the issues before where we have had transfer prisoners on the wards who are hard work, for whatever reason because their behaviour, and what tends to happen is people start saying to me well you know “why don't you just send them back to prison? They shouldn't be here, their causing trouble, you need to send them back” you know, which is a kind of a natural response when things aren't going well. But to my mind, especially if they're ill that can't be the right response, because you know presumably someone thought they needed to be in hospital in the first place because they're ill, if they're still ill and in hospital sending them back to prison isn't going to make them any better, it might make them worse. Whilst it might be hard work looking after them in hospital that's what you're there to do, I would have thought. I, I mean for me, I would only consider sending someone back to prison if they were well enough that they didn't need hospital and I realistically thought they would do well enough in prison. If I thought they were going to be, you know, too ill to go to prison, I wouldn't send them you know.’ P3, psychiatrist, MSS-based
	‘Okay if someone is a risk to the medium-secure unit, should we equally make an argument that they're a risk when they're in the prison environment?. I don't buy that as a reason for discharge, I have come across a few cases. There was one case recently of a guy who was transferred back to us … [information omitted due to sensitivity] … the next day with no CPA [Care Programme Approach], no handover, nothing and I thought that was poor actually because they were discharged back but also the same time a recommendation was made for an admission to maximum security. So I think you can't be on the one hand saying that somebody needs conditions of maximum security and needs to be in hospital but in the mean time they should be admitted to prison to wait for it. I think that was poor practice actually so it does happen around about risk, around about the ways of managing risk in medium security but I'm not sure that it should.’ P2, psychiatrist, prison-based
	‘We get in the discharge is discharge summary of the communications: “this person wouldn't engage, this person wouldn't engage with anything so they're coming back. They're taking antipsychotics, but they didn't engage in psychology so there you go”. Whereas I think well there are quite a lot of people in medium security who don't engage but you don't send them into the community do you? You don't go “oh well, then there you go”. They stay and they keep them in long term, you accept that it's going to be a long time, maybe send them to a long-term medium secure, but you don't just bounce them back to prison and it's almost like “well what do you think we're going to do in prison?” Is it somehow that they're safe in prison? Is it like a pseudo-hospital? Because it's not… it's almost like prison is a safe place, which is so far from the truth.’ P4, psychiatrist, prison-based
**Nature of mental disorder and services available**	
	It's slightly different for the mentally ill – the normal business, as we call them, which is stigmatising if anything, but with the personality disorder cases I think that the thinking is different. It's like we're saying, “if you're mentally ill we can give you treatment whether you want it or not. If you're personality disorder, you've got to be buying into it at some level to have treatment”. If you're mentally ill, and you're fighting against your treatment, you're more likely to stay but if you're personality disorder and you're fighting against your treatment, you're likely gone, because you're not engaging with it.’ P10, nurse, MSS-basedd
	‘I don't know whether it's the nature of the illness or what, the kind of relationship you develop with the patient when someone's acutely unwell and you're trying to get them better and then when you do get them better I think that there's a tendency to want to look after them a little bit – not more that's the wrong, not more, I don't really mean it like that but, I think there's just that nurturing to keep them well, where maybe there's not that [with personality disorder]. I think when you work with personality disorder I think sometimes it tend to be “well we've identified what the problem is so we'll work on that somewhere else”.’ P7, nurse, MSS-based
	‘But, of course, in prison, you're, the regime is much more about punishment and reward and boundary setting which of course often is the right kind of approach in personality disorder and that's harder to do in hospital where you've got a therapeutic regime. So it's not really based around rewards and punishments and times and stuff, its more based around sort of therapeutic goals and getting well, and of course, sometimes if you've got people with personality disorder they're not likely to get any better so if your regime is based on people getting better and you've got people who aren't going to get better then they're disadvantaged.’ P3, psychiatrist, MSS-based

MSS, medium-secure services.

## Results

Key findings are described in text and extracts from anonymised participant transcripts are provided in support of each insight in Tables 2–4. The study generated a vast amount of data, which is explored and presented under three distinct themes: theme 1 focused on the maintenance of throughput and service provision, theme 2 examined the clash of two systems and theme 3 focused on desirable and undesirable patients.

### Theme 1: maintenance of throughput and service provision

Theme 1 captures the policy influences perceived to drive the pressure on service throughput (i.e. ‘patient flow’ between MSS and the prison estate). Clinicians described the constraints present in the care and management of those admitted from prison, and how they perceive and respond to these. This included detailed discussion of commissioning pressures, bespoke service models and how reduced access to aftercare upon prison return shaped pathway decisions for some individuals admitted from prison.

#### External priorities and expectations

MSS-based clinicians described how commissioner requirements shape their discharge decision making and affect their clinical autonomy. Perceived pressure for MSS to receive admission from prison and length-of-stay targets were discussed, with length of stay described as a key performance indicator. Clinicians described a ‘circular’ care pathway for those admitted from prison, where the aim is to deliver treatment in the shortest possible period of time, followed by return to prison. As a result, ‘optimised provision’ was described, where, in some regions, new ward models now exist that act as a dedicated pathway for prison admission, with a quick turnaround. Although deemed appropriate for many admitted from prison, some clinicians described how this approach may not adequately take into account an individual's psychiatric diagnosis or best interests. This requirements/best interests conflict was described as mediated by availability of beds. For example, clinicians described scenarios where they had returned patients to prison too early so that they could admit another patient from prison. Many expressed concern that this may become standard practice, and highlighted that this could be a ‘dangerous’ solution to bed shortages. As such, there were also instances where clinicians described patient need outweighing their prison transfer status, with clinicians’ subsequently enforcing their clinical autonomy to continue in-patient care.

#### PMHS provision

Many MSS-based clinicians described instances where they had been reluctant to, or have opted not to direct prison return, to ensure discharge via a community care pathway and aftercare delivered through community mental health teams (CMHTs). Receipt of aftercare via a CMHT was described as more beneficial to that available in the prison estate. PMHS were described as both underfunded and poorly resourced, and clinicians shared previous negative experiences of patient outlook upon return to prison. These included examples of how the prison environment is detrimental to vulnerable prison returners, alongside a lack of access to mental health professionals. Many expressed concern that services post-return are not yet able to offer care that is equivalent to that provided by CMHTs, and that there are currently no resources or targeted support for those returning to prison from a psychiatric in-patient stay. These concerns extended beyond prison-based aftercare provision, into access to care at community release from custody. Continuity of care between PMHS and CMHTs upon release was described as poor compared with aftercare arrangements on discharge from MSS.

### Theme 2: clash of two systems

Theme 2 centres on the integration of the mental health and criminal justice system, specifically: how some admission and pathway decisions are at the discretion of the criminal court, and the ways in which court-based decisions and the patient's legal status can affect the RMO's authority to direct an individual's care pathway.

#### Patients who are on remand and pre-sentenced patients

Patients who are on remand can be transferred to MSS for psychiatric treatment by warrant of the Secretary of State (Section 48/49 MHA). Individuals will be detained in MSS until either the RMO thinks that they no longer require treatment and directs their return to prison, or until the individual's criminal case has been decided by the court. Additionally, when the issue of psychiatric diagnosis arises during the trial process, criminal courts are required to consider the ‘most suitable method of disposing of the case’ before passing a custodial sentence. This can involve remanding defendants for assessment (Section 35 MHA) and treatment (Sections 36 and 38 MHA) to inform sentencing decisions. The court is required to consider the medical evidence provided by the MSS clinical team before passing a custodial sentence.

Clinicians described instances of treating those transferred on remand (Section 48/49 MHA) who had been sentenced mid-treatment, and either released into the community or returned to prison, despite their detention in MSS. Clinicians also recounted other examples of how court disposal decisions had been made in opposition to their clinical recommendations, which had subsequently limited the discharge options available to the clinical team for these individuals. For example, instances where they had recommended a hospital treatment order, yet the court had opted to pass a custodial sentence. In these circumstances patients are often automatically returned to prison from court and have to be re-referred for admission as a sentenced prison transfer. Likewise, clinicians described examples of when the MSS psychiatric report had conflicted with the report provided by the court-appointed independent psychiatrist, causing the court to direct a re-admission for further assessment, or to order detention for treatment in lieu of a custodial sentence. Clinicians expressed their frustrations in response to these scenarios, as the court-imposed legal status limits their ability to shape the most appropriate treatment and discharge pathway for an individual. It was clear that these issues varied across both individual cases and regional areas.

#### Patients with a custodial sentence

Sentenced prisoners can be transferred to MSS for psychiatric treatment by warrant of the Secretary of State (Section 47/49 MHA). Their remaining sentence tariff governs whether discharge into the community at time of treatment completion is an option, unless their sentence lapses during their psychiatric detention, in which case community discharge is at the discretion of the responsible clinician.

All clinicians discussed to some degree the ways in which the remaining tariff on a patient's sentence can affect both the timing of discharge and the discharge destination. Clinicians described how those with particularly long outstanding sentence tariffs often require return to prison, as the service cannot justify continuing treatment for the remainder of their custodial sentence. At times, this was described as resulting in ‘revolving door cases’; patients who have multiple admissions to MSS during their custodial sentence, because of stopping medication or becoming reactively unwell once back in prison. This prompted consideration of how to best manage and prevent this, with some suggesting it may be more advantageous to keep individuals detained once readmitted. There were, however, described instances of MSS retaining patients with lengthy remaining tariffs because of the clinicians’ belief that these patients should have received a hospital treatment order at sentencing. It was described how these decisions ensure effective transition into the community and receipt of targeted aftercare.

A breadth of clinical opinion and experience was uncovered when discussing patients who have short amounts of time remaining on their tariff, or are close to their earliest release date. Discharge into the community from MSS was described as often slow and difficult to achieve. As such, return to prison was described as a quicker route into the community for sentenced patients who are close to their earliest release date. This was often at the patient's request, to avoid being ‘stuck’ in a MSS pathway, with some patients presenting as ‘unmanageable’ to prompt a quicker return. Retention of these individuals was described as impractical, particularly when patients do not wish to engage with the treatment process. In contrast, some clinicians were strongly opposed to this practice, and described how it is in the patient's best interest to remain in hospital to ensure adherence with medication and full treatment completion before community discharge/release. For this reason, clinicians also described how a sentence lapse during admission can be advantageous, as the patient's legal status will convert to a notional hospital order (Section 37N MHA), which results in the patient's discharge being solely at the discretion of their RMO. This was described as a means to ensure facilitation of necessary follow-up support, which they believed could not be ensured if these patients were returned to prison and promptly released into the community.

#### Patients with an indeterminate sentence for public protection

The imprisonment for public protection (IPP) sentence was a form of indeterminate sentence in which offenders were given a minimum prison tariff, but no maximum, for a range of crimes. For patients who are subject to an IPP sentence, release into the community is at the sole discretion of the prison-based parole board; therefore, the discharge pathway following treatment is return to prison. Once returned, individuals are required to satisfy particular parole board conditions, such as completing offender management programmes to demonstrate a reduction in the risk they pose, to secure a release date.

The treatment and discharge of those serving IPP sentences was described as problematic. Clinicians described patients that they believed were ‘stuck in the system’, who are ineligible for RMO-directed discharge into the community, but release into the community upon return to prison is not guaranteed. Many described how individuals who completed ‘violence reduction programmes’ within MSS were still required to return to prison to complete similar but accredited modules; however, these courses are not always available at the receiving prison. There was a collective frustration described by clinicians when an individual is deemed suitable for discharge into the community from MSS, and the morality of these sentences was questioned.

### Theme 3: desirable and undesirable patients

Themes 1 and 2 centre around the ways in which clinical discharge decisions can be shaped by external expectations, service provision or the constraints imposed by court sentencing decisions. Theme 3 encapsulates the discretionary pathway decisions that MSS make when prioritising patients for continued in-patient treatment. This includes discussion on how clinicians view the role and function of MSS within the wider forensic mental health system, and therefore the types of patients that are deemed ‘appropriate’ for the service.

#### Forensic patients versus acutely unwell prisoners

The nature of an individual's psychiatric diagnosis and its relevance to their offending behaviour were described as key determinants of their appropriateness for continued care and supported discharge via a community care pathway. This absence of a relationship between psychiatric diagnosis and offending, and therefore the degree to which the patient is deemed to be culpable for their offence, was also described as eliciting a punitive response within clinical teams.

Prison transfers were viewed as ‘offenders’ who have become acutely unwell in prison, whereas patients who were subject to hospital treatment orders were viewed as those whose diagnosis and offending is linked, and were therefore described as ‘true forensic patients’. For forensic patients, the role of MSS was described as to provide treatment, rehabilitation, offence-related risk reduction and reintegration into society via a community care pathway, whereas for prison transfers, offence-related or risk reduction work was deemed neither necessary nor the responsibility of MSS. Although the individual may pose public protection concerns at the time of prison return, MSS-based clinicians described that it is the role of the prison estate to contain and address this risk before community release. Prison-based clinicians, however, described how these risks are not guaranteed to be addressed by the prison estate upon return. It was described that PMHS do not have the resources to implement the required risk reduction work before release. The outcome for these patients is uncertain, with some going on to offend and re-enter custody. One clinician suggested that these attitudes were evidence of an institutional effect within the MSS estate. Services were described as increasingly ‘looking inward’ and setting boundaries to protect their function (as they view it) within the wider forensic mental health system.

#### Nature of in-patient presentation

*‘*Undesirable’ presentation, such as treatment non-adherence/disengagement and high-risk behaviours, were described as contributing to the ‘success’ of the treatment phase for those admitted from prison, in terms of length of the individual's admission and their subsequent care pathway. Successful patients were described as those who comply with their medication/intervention, for whom clinicians are less likely to consider prison return if they are also engaged in the therapeutic interventions outlined in their care plan. For these patients, opportunity for a longer admission was available and discharge via a community care pathway was described as more likely. It was described that return to prison of non-engaging patients is, at times, conducted with the intent to free a bed for other prison-based patients. Clinicians explained that this also avoids sentence lapse during detention, whereby the MSS may be ‘stuck’ with a non-engaging patient. Not all MSS-based clinicians endorsed this practice, with some championing how it is the role of MSS to work holistically to ensure that patients complete all necessary treatment, regardless of difficult presentation. These clinicians also highlighted their concerns regarding patient outlook upon prison return under these circumstances. These concerns were shared by prison-based clinicians, who provided examples of the difficulties faced upon receipt of non-engaging patients.

High-risk behaviour, such as violence toward staff members, was also presented as a common reason for return to prison. In these circumstances, ward security and the safety of clinicians and other patients was deemed a priority, regardless of the patient's engagement with treatment. MSS-based clinicians felt that it is the role of the prisons and not the MSS to manage this behaviour, and a shift in overall risk responsibility was described. It was felt that return of these patients is necessary not only to protect the nursing staff, but to also protect and delineate the role and function of MSS. Prison-based clinicians described how in these circumstances, prison was being judged inappropriately as a ‘safe’ discharge destination, where the ‘correct’ response should be referral to high-secure hospital.

#### Nature of diagnosis and services available

MSS-based clinicians described how the clinical approach and tolerance of unmanageable behaviour was dependent on the patient's primary psychiatric diagnosis. Although it was recognised that patients with a primary diagnoses of SMI may cause ward disruption and present as unmanageable, the clinical response was described as caring and nurturing, whereas approach for those with a primary diagnosis of a personality disorder was described as punitive. In these circumstances, return to prison was described as more advantageous for those with a personality disorder, and the regimented nature of prison was described as more suited to their needs. Overall, there was consensus across MSS-based clinicians that MSS are not an appropriate environment for managing those with a personality disorder. Some MSS-based clinicians described how identification of a personality disorder diagnosis upon admission can lead to a return to prison on that basis alone. Others stated that they would not admit a patient from prison to their service that had a primary diagnosis of a personality disorder and no secondary diagnosis, as prison was believed to be a more appropriate environment than attempting to admit a patient to a service that has no treatment provision for personality disorders.

Although some dedicated services exist, there is currently little national provision within the MSS estate that is designed for the treatment of personality disorders. Clinicians described how these services currently operate to tight admission criteria, and are notoriously difficult to negotiate admission to. These services were described as for individuals with a personality disorder who are ready to engage in the services and complete the required piece of work. However the discharge pathway out of these services remains return to prison post-treatment. As such, concern was expressed about the limited services available for these patients post-return, with prison-based clinicians sharing their concerns about the lack of care and treatment for personality disorders within the prison estate.

## Discussion

There is wide variation in available resources to manage MSS, and many different styles of service delivery exist.^16^ For the majority of admissions, services are required to provide assessment and/or treatment, rehabilitation and management of the risk that patient poses to others, with the view to reducing reoffending.^2^ This involves undertaking clinical and risk interventions, followed by safe discharge of patients to lower levels of security, back into the community or back to prison. Therefore, in our previous study, it was unclear why people were returned to prison after short lengths of stay and/or because of being close to their earliest release date, treatment non-engagement or presenting as too high risk.

The present study highlights an array of factors that could restrict a clinician's autonomy when making pathway decisions, such as custodial sentences, court disposal decisions, when the remaining sentence tariff makes prolonged admission unfeasible or when release is solely at the discretion of the Ministry of Justice. Likewise, gate-keeping responsibilities and maintenance of service throughput were, at times, deemed to underpin prison return decisions. Clinicians described instances where they compromise patients’ best interests to satisfy commissioning criteria, particularly in relation to reducing lengths of stay and ensuring prison return. Concern was raised that the quality of care these individuals receive post-return is not equivalent to CMHT-delivered care. Despite these concerns, these factors also influence discretionary pathway decisions. Across MSS-based clinicians, there was a clear drive to protect the remit of their service, as they viewed it. Admission from prison and prolonged length of stay was described as a ‘valuable opportunity’, and characteristics of ‘appropriate’ patients were proposed. The degree to which treatment non-engagement and high-risk behaviours were tolerated was described as dependent on an individual's primary diagnosis, where those with a personality disorder were more likely to be remitted to prison on this basis. This is consistent with earlier findings that prison returners are characterised as more likely to have a personality disorder diagnosis and higher risk of future violence, when compared with those discharged into the community.^11^ The reluctance for MSS to accept admissions that have a primary personality disorder diagnosis has been documented within the literature for over a decade.^8,9^ If secure and prison-based mental health services are to continue to function under the current nexus, then consideration should be given to the function and further development of targeted resources for prisoners with personality disorder diagnoses, such as the Offender Personality Disorder (OPD) pathway – a jointly commissioned initiative between mental health services and the criminal justice system.^17^ It is estimated that there are some 30 000 prisoners who would benefit from the OPD programme, of whom just a fraction are able to access the service at present. Prisoners with a personality disorder are therefore disadvantaged both within MSS and the prison estate. Further resources are required to meet the clinical needs and interventions that may benefit these individuals, with a significant expansion of the OPD pathway likely required.

Overall data from this study revealed that there remains a deficit in adequate resources and treatment to meet the mental health needs of prisoners, both within prison and secure services. It has previously been suggested that provision should be made for longer treatment periods for those admitted from prison, and perhaps retention of more individuals until sentence completion, to ensure discharge via a community mental health pathway.^11^ Although this may ensure more adequate transition into the community, it may also result in prolonged treatment beyond sentence tariff, raising both ethical and legal issues. As such, it is unlikely that this suggestion is practical at present, as although transfers from prison to secure services are increasing,^18^ this is just one admission source for MSS. Secure services also admit those sentenced to hospital treatment orders and are ‘step-up/-down’ services for high- and low-secure services. As such, to meet the clinical need of all admissions, provision for the MSS estate will need to increase substantially. In a time of economic constraint, it is unclear how feasible this is. Alternative models of PMHS have been proposed, including the contentious topic of designated ‘prison hospitals’,^19,20^ as are present across areas of Europe and the USA.^21–23^ However, there are ethical implications for implementing services of this type. It is well-established that the prison environment itself is subtherapeutic, and there remain conflicting priorities between security and healthcare; for example, inappropriate practices of placing seriously mentally ill prisoners in segregation cells, and the excessive use of control and constraint measures.^24^ Nevertheless, the integrated support unit (ISU) model is currently being piloted within the UK prison estate, which appears to act as a buffer to manage hospital transfer delays, as opposed to a prison hospital. It has been reported that this provision allows some individuals to recover before a hospital transfer. In these cases, individuals are transitioned back into the general prison population; however, there is little description of follow-up and aftercare provision.

Regardless of new innovation, it remains the case that there is not adequate provision of PMHS to meet the current level of clinical need. If the described ‘circular pathway’ is to continue, it is clear that there is a requirement for a fully funded PMHS, which is integrated with local secure psychiatric services – a stance shared by all participants in this study. Throughout the interviews, participants were extremely honest about the current issues faced and difficult decisions that are required of them. At present, there is no national forum for service planning and discussion of these important issues for clinicians. We propose that such a forum needs to be made available as a place to share good practice and to gain feedback on service initiatives.

### Limitations

Although we interviewed an eclectic group of clinicians, our sample composition is not without bias. For both the individual interviews and focus group, we identified clinicians who met our criteria when conducting our previous study.^12^ These were clinicians that we knew worked on a remittal care pathway and were therefore invited directly by email. Likewise, although the convenience sampling strategy ensured focus group attendance, the characteristics of the conference attendees may have biased the findings. Those in attendance were likely to be professionals who were engaged in research and knowledgeable of the key policy pertinent to the study. Attendees may not be representative of the average forensic psychiatrist; therefore, this should be acknowledged when reading and drawing conclusions from the focus group data. Additionally, although professionals from outside of psychiatry were invited to take part, just one social worker agreed. Psychologists were invited to take part based on their expertise in risk assessment, although none of the psychologists who were approached to take part agreed to be interviewed. Likewise, nursing staff from mental health in-reach services were also invited to take part, to provide the nursing perspective on issues raised by prison-based psychiatrists. There was little uptake from prison in-reach nurses, and the two interviews that were arranged failed to go ahead because of staff shortages on the day of interview. It is unfortunate that the clinicians from professions other than psychiatry were not adequately represented for the purpose of this study. Therefore, this should be acknowledged when reading and drawing conclusions from the interview data.

## Data Availability

The authors maintain sole access to the study data. Access is ongoing while further analysis is taking place.

## Supplementary material

For supplementary material accompanying this paper visit http://dx.doi.org/10.1192/bjo.2021.928. click here to view supplementary material
